# Study on the Oxygen Enrichment Effect of Individual Oxygen-Supply Device in a Tunnel of Plateau Mine

**DOI:** 10.3390/ijerph17165934

**Published:** 2020-08-15

**Authors:** Zijun Li, Rongrong Li, Yu Xu, Yuanyuan Xu

**Affiliations:** School of Resources and Safety Engineering, Central South University, Changsha 410083, China; zijunli@csu.edu.cn (Z.L.); rongrongli@csu.edu.cn (R.L.); xuyuanyuan@csu.edu.cn (Y.X.)

**Keywords:** plateau mine, individual oxygen enrichment, numerical simulation, orthogonal design, range analysis

## Abstract

Complex characteristics of the plateau environment such as low oxygen content seriously restrict the exploitation of abundant mineral resources in plateau areas. To regulate the hypoxia environment and improve the comfort of workers engaged in intense physical labor like tunnel excavation operations in plateau mines, an individual oxygen-supply device for tunnel of plateau mine was proposed to create local oxygen enrichment in the area around the human nose. The Computational Fluid Dynamics (CFD) method was used to judge the application’s effect of the individual oxygen-supply device in plateau mine, revealing the oxygen diffusion law under the influence of different oxygen enrichment factors. The orthogonal design and range analysis were used to measure the degree of influence of major factors such as oxygen-supply velocity, oxygen-supply concentration, and tunnel airflow velocity. The results demonstrate that the oxygen mass fraction of the air inhaled by the human had a positive correlation exponential function, a positive correlation linear function, and a negative correlation exponential function, respectively, concerning oxygen-supply velocity, oxygen-supply concentration, and tunnel airflow velocity. The range analysis revealed that the major influencing factors of oxygen enrichment in the tunnel of the plateau mine were, in a descending sequence, as follows: oxygen-supply concentration, tunnel airflow velocity, and oxygen-supply velocity, and the corresponding ranges were 2.86, 2.63, and 1.83, respectively. The individual oxygen-supply device achieved the best oxygen enrichment effect when the oxygen-supply velocity was 5 m/s, the oxygen-supply concentration was 60%, and the tunnel airflow velocity was 0.2 m/s, which increased the oxygen mass fraction of air inhaled by the human to 30.42%. This study has a positive guiding significance for the improvement of the respiration environment in the tunnel of plateau mine.

## 1. Introduction

The western plateau of China is rich in mineral resources and belongs to an important reserve base for mineral resources because of its complex geological structure and superior mineralization conditions. With the further development of the western region and the deepening of the Belt and Road Initiative, the development of plateau mineral resources in western China has been carried out in an orderly manner. The safety of mining operations and the health of miners in the plateau are affected by the complex and diverse plateau climate environment, including low air pressure, low oxygen content, and large temperature difference. Among them, the problem of low oxygen content is particularly prominent, which seriously affects the health and labor efficiency of miners [1,2]. The volume percentage of oxygen is independent of the increase in elevation. However, air density decreases in high-altitude areas, resulting in a lower oxygen partial pressure and affecting human health [3].

Extensive research has been devoted to investigating the impact of low-oxygen environments on people’s physical health in the plateau area in both physical and psychological aspects. In terms of physiology, scholars mainly study the effects of hypoxia on the human body from various basic physiological indicators. Valant [4] studied the effect of oxygen concentration on blood flow characteristics and found that low oxygen concentration will decrease blood viscosity. Osculati [5] discovered that healthy subjects exposed to hypoxia may develop subendocardial contractile dysfunction by recording physiological and echocardiographic variables. Koehle [6] found that subjects with a blood oxygen saturation above 86% are more likely to be free from acute high-altitude disease by evaluating the heart rate, oxygen saturation, and blood pressure. Vinniko [7] found that working at high altitudes may accelerate the decline in lung function, but the rate of decline in lung function combined with other factors requires further research. Lawley [8] pointed out that acute high-altitude disease can lead to an increase in individual brain volume and intracranial pressure. In psychological terms, Boos [9] explored the relationship between anxiety and risk of the acute high altitude disease, suggesting that anxiety in high-altitude areas was independently associated with acute high-altitude disease, while Sracic [10] expressed that hypoxia was a cause of anxiety symptomatology of the environment. Pavlicek [11] discovered that cognitive flexibility and affective functions remained unchanged under the condition of functional impairment of the vasomotor center due to central hypoxia. In addition, some scholars have proven from various physiological indicators of the human body that medical treatment [12,13] and oxygen-supply [14,15] can effectively alleviate the impact of hypoxia on human health. However, the use of medical treatment is limited, and it is not suitable for people who work in the plateau area for a long time. Therefore, in order to alleviate the hypoxic problem of workers engaged in high-intensity manual labor in the plateau area, the study of artificial oxygen-supply technology is of great significance.

At present, various types of oxygen-supply methods and related technical devices have made some progress, also applied in an advanced way in the fields of plateau tunnel ventilation [16,17], enclosed space [18,19], aviation technology [20,21], and medical institutions [22,23]. The research on the artificial oxygen-supply technology for the plateau area mainly focused on the oxygen production method and the optimization design of the oxygen enrichment device. The portable oxygen enrichment device for individuals [22] improved the previous oxygen-increased respirator [24] using oxygen enrichment membrane to obtain a suitable concentration of oxygen, which can not only increase the level of SpO_2_ and physical capacity but also reduce the heart rate. The hyperbaric oxygen chamber [25] is capable of compressing air pressure in the oxygen chamber to three times absolute atmospheric pressure and can be used to treat acute mountain sickness. Oxygen distributing equipment [26] sprays oxygen out of its multiple emission holes in the form of jet atomization, forming an oxygen curtain. Two sets of oxygen curtains will occur when the two groups of oxygen distributors are arranged relatively, thus generating a local oxygen-rich area with high oxygen concentration. Low-pressure swing adsorption system [27] used the low-pressure swing adsorption method to improve oxygen production efficiency, and successfully applied in the development of the Qinghai–Tibet railway engineering project, which enhanced the PAO_2_ of the tunnel section by 2–3 kPa.

Despite the considerable amount of research performed by the aforementioned scientists, their studies mostly focused on the oxygen-supply methods related to the construction of plateau highway tunnels, and few researchers systematically explored how to alleviate the effects of low oxygen content on workers in the tunnel of plateau mine; additionally, physical models established in the current studies using numerical simulation does not incorporate the human body model, ignoring the influence of the human body on the oxygen supply effect. Moreover, the way of large-scale dispersion oxygen supply is adopted in the tunnel of plateau mine, resulting in serious oxygen loss and low effective utilization rate. The individual accurate oxygen-supply mode, by contrast, can significantly improve oxygen utilization rate, and make the control of oxygen release quantity more convenient, which enables precise control of the breathing environment.

In this study, an individual oxygen-supply device was proposed to specifically supply oxygen to the area around the human nose through a nozzle, thereby improving the utilization rate of oxygen and the breathing environment of underground workers with lower energy consumption. Firstly, the Computational Fluid Dynamics (CFD) method was used to explore the optimal nozzle arrangement of the individual oxygen-supply device. The corresponding numerical model was established in the COMSOL Multiphysics software (COMSOL Inc., Stockholm, Sweden), and the boundary conditions were determined based on the actual conditions of the tunnel environment of Pulang copper mine, at an altitude of 3400 m in Yunnan, the southwest of China. Furthermore, the influence of oxygen-supply velocity, oxygen-supply concentration, and tunnel airflow velocity on the stability of the device was analyzed based on the optimal nozzle exit position. Finally, orthogonal design and range analysis were used to compare the importance of oxygen enrichment factors. This research can provide theoretical and technical guidance for improving the respiratory environment of mine tunnels at high altitudes.

## 2. Individual Oxygen-Supply Device System Description

**Figure 1:**
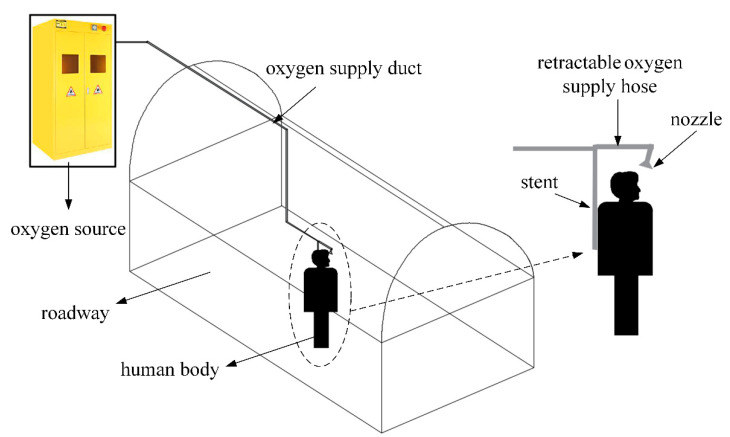
Diagram of individual oxygen-supply device.

The local oxygen enrichment method proposed in this paper can supply oxygen centrally at the target position, which is different from the conventional tunnel diffusion oxygen-supply method. The individual oxygen-supply device supplies oxygen specifically for the area around the human nose, thereby improving the breathing environment of underground workers. The individual oxygen-supply device is shown in Figure 1. The availability of oxygen sources in the plateau area is restricted by many factors, such as reserves, traffic conditions, meteorological conditions, transportation distance, etc., and the use of individual oxygen-supply devices can effectively overcome the problem of the limited oxygen source and improve the effective utilization of oxygen. The individual oxygen-supply device was mainly composed of three parts: oxygen source, oxygen-supply duct, and oxygen-supply nozzle. Details are as follows:

(1) The oxygen delivered to the working area through the oxygen-supply duct stems from the oxygen source formed by the oxygen cylinder and the gas cylinder cabinet.
(2) The oxygen-supply nozzle was fixed diagonally above the head of the human body through the stent, and the oxygen-supply nozzle outlet is inclined to the side of the nose.
(3) The oxygen-supply duct and the oxygen-supply nozzle are connected by a retractable oxygen-supply hose that can make oxygen supply duct and nozzle to maintain connection when worker moves.

The high concentration of oxygen sprayed from the nozzle makes the region of the human breath rich in oxygen, thereby achieving the purpose of improving the human respiratory environment. This method is easy to implement in a complex mine environment. Compared with the oxygen-supply in a large area in the tunnel, it can greatly reduce the amount of oxygen needed to increase oxygen and reduces the cost of oxygen-supply.

## 3. Modeling and Simulation

### 3.1. Physical Model

**Figure 2:**
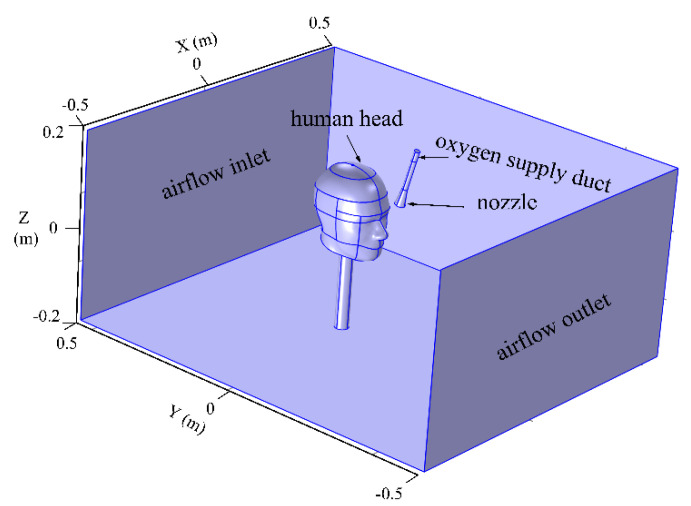
Physical model of individual oxygen-supply device.

Based on the above contents, the calculation model of the human body, individual oxygen-supply device, and local environment of mine tunnel were established in the COMSOL Multiphysics software (COMSOL Inc., Stockholm, Sweden) to judge the application effect of the individual oxygen-supply device in plateau mine, as shown in Figure 2. Among them, the size of the developed model was 1 m long, 1 m wide, and 0.4 m tall, and a 4.76 × 10^−4^ m^2^ intake port was designed at the nostril of the human body for oxygen inhalation. Moreover, the oxygen-supply duct was 0.008 m in diameter, and the nozzle was tilted 15°C towards the mouth of the human body, with a diameter of 0.03 m.

**Figure 3:**
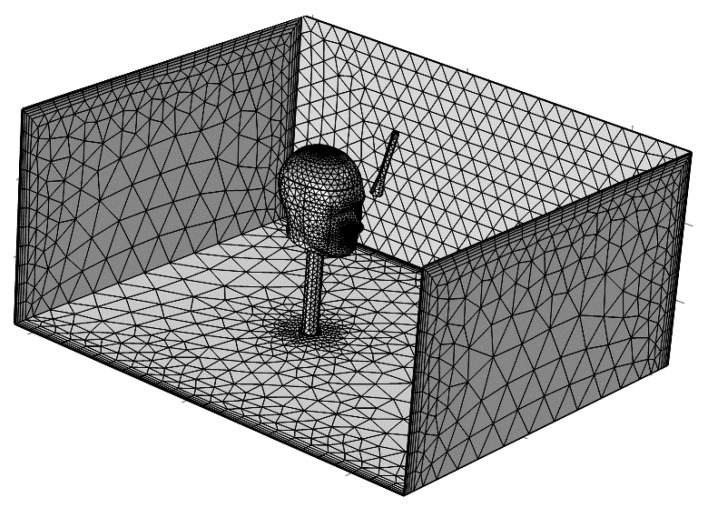
Generation of tunnel grid.

Considering that there were ventilation airflows in the tunnel, a stable airflow inlet and outlet were respectively set at the back end and front end of the model to study the influence of the tunnel airflow on the local oxygen enrichment. In order to improve the accuracy of the model calculation, the mesh of the head area and nozzle area were refined locally. As a result, the total mesh number of the model was up to 70,000. The geometric model grid after optimization was shown in Figure 3.

Based on above, three different nozzle outlet positions were analyzed based on the oxygen enrichment effect to obtain the best layout of the oxygen-supply nozzle. All other conditions were the same in each calculation model, and the oxygen enrichment effect was analyzed in the following cases: (1) In Case 1, the level of the nozzle outlet was lower than 0.06 m lower than the top of the head, and the oxygen-supply duct was vertical down. (2) In Case 2, the level of the nozzle outlet was level with the human head, and the oxygen-supply duct was tilted 15 °C times towards the human nose. (3) In Case 3, the level of the nozzle outlet was 0.06 m lower than the top of the head, and the oxygen-supply duct was tilted 15 °C times towards the human nose.

### 3.2. Boundary Conditions and Turbulence Model

**Figure 4:**
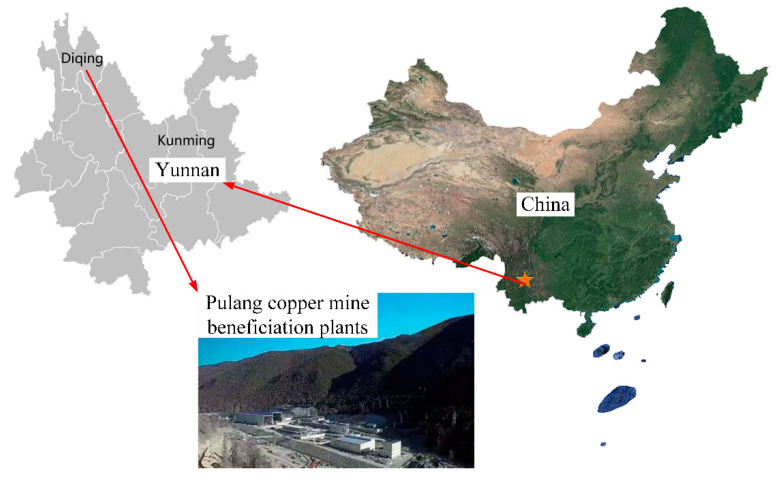
Location of the Pulang copper mine.

The CFD model was built based on Pulang copper mine in Yunnan, the southwest of China, which is currently the largest porphyry copper mine found in Asia. It is located between 3450 m and 4500 m above sea level, and obvious low pressure and hypoxia problems are common. The specific location of the mine was shown in Figure 4. Taking the blind heading space of Pulang copper mine as an example, boundary conditions were set. The mining area belongs to subtropical mountain monsoon climate, with an annual mean temperature around 4 °C, average summer temperature around 15 °C, and average winter temperature around −5 °C. The average summer temperature 15 °C (288.15 K) was taken into consideration in the design of the simulation model.

Atmospheric pressure was calculated from an empirical formula for pressure and altitude, as shown in (1).

(1) $$P_{H}=101.325\times {\left(1-\frac{H}{44329}\right)}^{5.255879}$$

where H is the height above sea level, m, and P_H_ is the corresponding atmospheric pressure, expressed as absolute pressure, Kpa.

It can be calculated from Equation (1) that the atmospheric pressure at 3400 m above sea level is 66.614 Kpa.

Air density was calculated from the ideal gas law, as shown in (2).

(2) PM=ρRT

where P is atmospheric pressure, Pa; M is the molar mass of air, 29 g/mol; R is the proportional constant (for any ideal gas, R is constant, 8.314 J/(mol·K)); T is temperature.

It can be calculated from Equation (1) that the air density is 0.8064 kg/m^3^. In the model, the air is assumed to be incompressible fluid with a density of 0.8064 kg/m^3^. The boundary conditions of this study are shown in Table 1. Among them, the oxygen-supply flow rate is 1.0048 dm^3^/s, and the breathing volume at work is 127.8 L/min.

The k-Epsilon turbulence model has a good convergence rate, which is suitable for solving exterior flow problems with complex geometry and has been widely used in numerical simulation related to mine ventilation [28,29]. Due to the complexity of the geometric structure of the human body, the K-Epsilon model is adopted in this paper. K-Epsilon model considers two equation model that deals with turbulent kinetic energy, k, and its rate of dissipation, ε, which is coupled with turbulent viscosity. This model is given as [30]:

(3) $$\frac{\partial }{\partial t}\left(\rho k\right)+\nabla \cdot \left(\rho \mathrm{Uk}\right)=\nabla \cdot \left[\left(\mu +\frac{\mu_{t}}{\sigma_{k}}\right)\nabla k\right]{+G}_{k}-\rho \varepsilon$$

(4) $$\frac{\partial }{\partial t}\left(\rho \varepsilon \right)+\nabla \cdot \left(\rho U\varepsilon \right)=\nabla \cdot \left[\left(\mu +\frac{\mu_{t}}{\sigma_{\varepsilon }}\right)\nabla \varepsilon \right]{+C}_{1\varepsilon }\frac{\varepsilon G_{k}}{k}+C_{2\varepsilon }\rho \frac{\varepsilon^{2}}{k}$$

where G_k_ represents the generation of turbulence kinetic energy due to the mean velocity gradients, m^2^/s^2^; C_1ε_ and C_2ε_ are model constants; σ_k_ and σ_ε_ are the turbulent Prandtl numbers corresponding to the k equation and the ε equation, respectively; ρ is the density of the fluid, kg/m^3^; µ_t_ is turbulent viscosity given by:

(5) $$\mu_{t}=\rho C_{\mu }\frac{k^{2}}{\varepsilon }$$

The values of C_1ε_, C_2ε_, C_µ_, σ_k_, and σ_ε_ are 1.44, 1.92, 0.09, 1, and 1.3, respectively.

This paper aimed to study the effect of the individual oxygen-supply device under the steady-state of oxygen-supply, so the model adopted the steady-state calculation method.

## 4. Simulation Results

**Figure 5:**
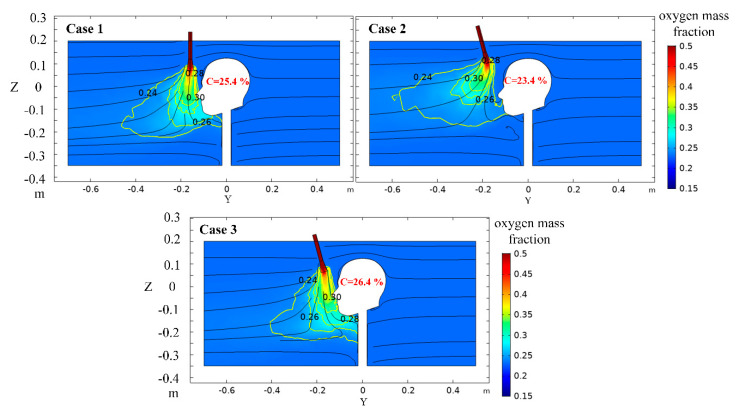
Distribution of oxygen mass fraction in the X = 0 m section.

The simulation results of the above three cases are shown in Figure 5; the relevant explanations are as follows:

(1) Case 1: when the oxygen was ejected vertically downward through the nozzle, the area with high oxygen mass fraction was located in front of the human nose. Moreover, the diffusion of oxygen far away from the human nose increased with the interference of the airflow in the tunnel, and the oxygen mass fraction of the air inhaled by the body was 25.4%.
(2) Case 2: the level of the nozzle exit of Case 2 was equal to the top of the head of the human body, which resulted in the reduction of the blocking effect of the human body on the airflow in the tunnel. Therefore, the disturbance of the airflow in the tunnel was greater when the oxygen of high concentration was ejected from the nozzle, a large amount of oxygen diffused to the front of the human body with the airflow of the tunnel. As a result, the effect of the individual oxygen-supply device was not obvious, and the oxygen mass fraction of the air inhaled by human increased slightly to 23.4%.
(3) Case 3, the angle of the oxygen-supply duct was 15 °C, and the nozzle outlet was aligned to the direction of the human nose. Due to the inertia effect, more oxygen was concentrated between the human nose and mouth, which improves the oxygen mass fraction of the air inhaled by human. Furthermore, the level of the nozzle outlet was 0.06 m lower than the top of the human head, which made the blocking effect of the human body on the airflow of the tunnel stronger, so the high concentration of oxygen emitted by the nozzle was less affected by the airflow in the tunnel, and the oxygen mass fraction of the air inhaled by human reached 26.4%.

According to the analysis, the position of the nozzle of Case 3 was the best. Therefore, the following simulation adopted the scheme of the angle of the oxygen-supply duct 15° and the level of the nozzle outlet 0.06 m lower than the top of the head.

**Figure 6:**
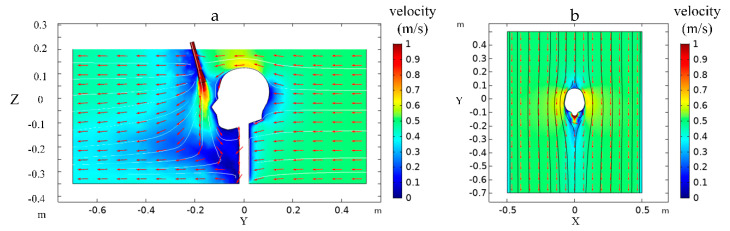
(**a**) Side view of air velocity at X = 0 m section. (**b**) Top view of air velocity at Z = 0.05 m section.

Figure 6 was the airflow distribution diagram of the tunnel when the nozzle position of Case 3 was taken. It can be seen from Figure 6 that the maximum velocity attenuates rapidly in the axial range after high concentration oxygen is ejected from the oxygen supply nozzle. This is because the oxygen-rich gas enters the environment through the nozzle exit and is affected by the ambient gas viscosity resistance in the axial direction. The flow velocity is very high within a short range from the nozzle exit, and the resistance is also very high, resulting in a fast velocity attenuation. However, under the action of the nozzle, the diffusion range of the air jet is enlarged, and a high-velocity flow area is formed near the human nose. When the airflow in the tunnel passes through the human head, the high-velocity area is generated on both sides of the human head and the top of the head, but the velocity in the human face area is smaller because of the obstruction of the human body to the airflow in the tunnel. Therefore, when the human body is facing away from the airflow in the tunnel, the airflow has little influence on the oxygen diffusion emitted from the individual oxygen-supply device.

**Figure 7:**
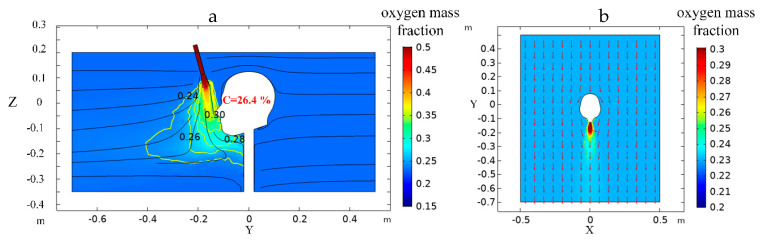
(**a**) Side view of oxygen mass fraction distribution at X = 0 m section. (**b**) Top view of oxygen mass fraction distribution at Z = 0.05 m section.

As shown in Figure 7, after the oxygen is ejected from the nozzle through the oxygen-supply duct, the oxygen mass fraction decreased gradually as the distance from the outlet of nozzle increased, which is consistent with the law that the maximum axial concentration of oxygen decays with the increase of the axial distance of the oxygen outlet in the case of diffusion oxygen-supply [31]. When using the nozzle to supply oxygen locally, the high oxygen mass fraction area mainly concentrates near the human nose, effectively enhancing the oxygen utilization ratio. In this case, the oxygen mass fraction of the air inhaled by human reached 26.4%, and the oxygen consumption was 0.06 m^3^/min. Compared with the traditional mode of directly using an oxygen-supply duct to increase oxygen in a wide range, the oxygen flow needed reached 12.46 m^3^/min when the oxygen mass fraction in the tunnel increased to 25.9% [32]. Therefore, the application of the individual oxygen-supply device has greatly reduced oxygen consumption and achieved a better oxygenating effect.

## 5. Stability Analysis of Individual Oxygen-Supply Device

The distribution of oxygen in the tunnel is closely related to factors such as oxygen-supply conditions and ventilation environment. Therefore, this study took into account the influences of oxygen-supply velocity, oxygen-supply concentration, and tunnel airflow velocity on the effect of oxygen enrichment, calculating the oxygen distribution under the action of different influencing factors. The oxygen enrichment stability of individual oxygen-supply device under different conditions was studied by taking the oxygen mass fraction of the air inhaled by the human and the regional volume where the oxygen mass fraction in the air in the tunnel increased by 5%, that is, the oxygen mass fraction reached 24.35% as the analysis index.

### 5.1. Oxygen-Supply Velocity

Oxygen-supply quantity is a key parameter that affects the local oxygen enrichment effect, and too little oxygen-supply quantity leads to poor oxygen enrichment effect, while too much oxygen-supply quantity causes a large amount of oxygen waste and increases oxygen-supply cost. In order to study the effect of different oxygen-supply quality, the oxygen-supply velocity was selected as 2 m/s, 4 m/s, 6 m/s, 7 m/s, and the corresponding oxygen supply was 0.003 m^3^/min, 0.006 m^3^/min, 0.009 m^3^/min, 0.011 m^3^/min, and the other setting conditions were consistent with Case 3.

**Figure 8:**
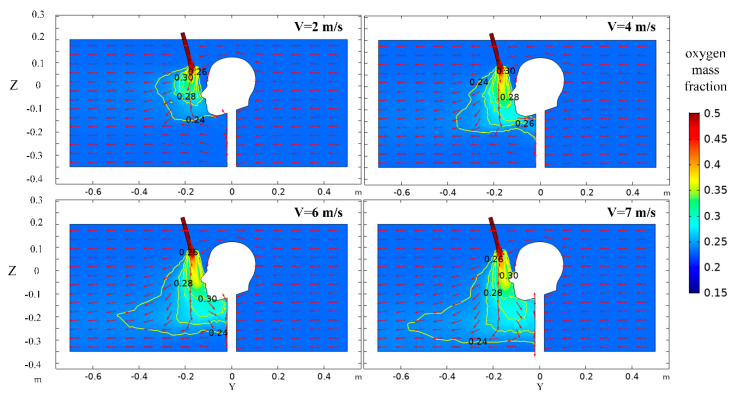
Distribution of oxygen mass fraction in the X = 0 m section at different oxygen-supply velocities.

As shown in Figure 8, with the increase of oxygen-supply rate, the diffusion range of oxygen increases significantly. The reason is that after oxygen is ejected from the nozzle, it has axial and radial ductility, and the larger the oxygen exit velocity is, the larger the oxygen enrichment range is [33], so the radial width and axial length of the oxygen enrichment region extend farther. However, despite the difference in oxygen-supply velocity, there is a large oxygen mass fraction gradient between the nozzle outlet and the human nose, which is due to the high flow rate of oxygen-rich gases within a very short distance of the oxygen outlet and is subject to great viscosity resistance of the surrounding gas, which makes the maximum velocity decay very fast, and the oxygen diffusion ability is weakened. When the oxygen mass fraction is reduced to 24%–27%, it enters a relatively stable decay process until it is close to the ambient oxygen mass fraction.

**Figure 9:**
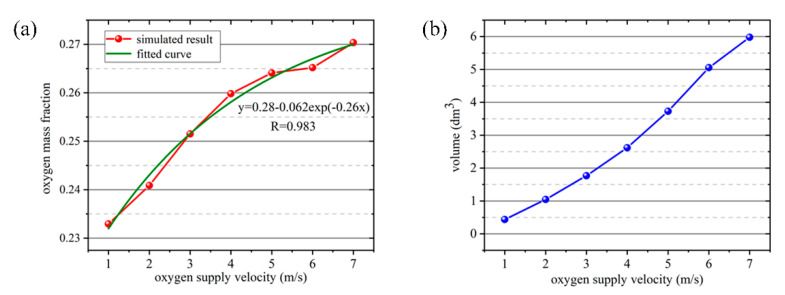
(**a**) Fitted curve of oxygen mass fraction of the air inhaled by human at different oxygen-supply velocities. (**b**) Volumetric distribution of regions with oxygen mass fraction up to 24.35% at different oxygen-supply velocities.

The oxygen mass fraction of the air inhaled by human at different oxygen-supply velocities was fitted as a function, and the results were shown in Figure 9a. It can be seen from the figure that the growth curve of oxygen-supply velocity and the oxygen mass fraction of the air inhaled by human is a monotone increasing function, according with the exponential function relationship. With the increase of oxygen-supply velocity, the increase of oxygen inhaled mass fraction of the human body is somewhat slowed down. In practical application, the oxygen-supply rate can be adjusted according to the engineering requirements to improve the oxygen mass fraction of the air inhaled by humans. As shown in Figure 9b, the volume of the region with the oxygen mass fraction reached 24.35%, gradually increasing with the increase of oxygen-supply velocity. when the oxygen-supply rate was greater than 6 m/s, the increased range was decreased.

### 5.2. Oxygen-Supply Concentration

The concentration difference is one of the main driving forces for the mixing of oxygen-supply and ambient air, which has an important influence on the distribution of oxygen-supply in the tunnel. To study the effect of different oxygen concentrations on local oxygen enrichment, the oxygen concentration of 40%, 50%, 60%, and 70% were selected in the calculation, and other setting conditions were consistent with Case 3.

**Figure 10:**
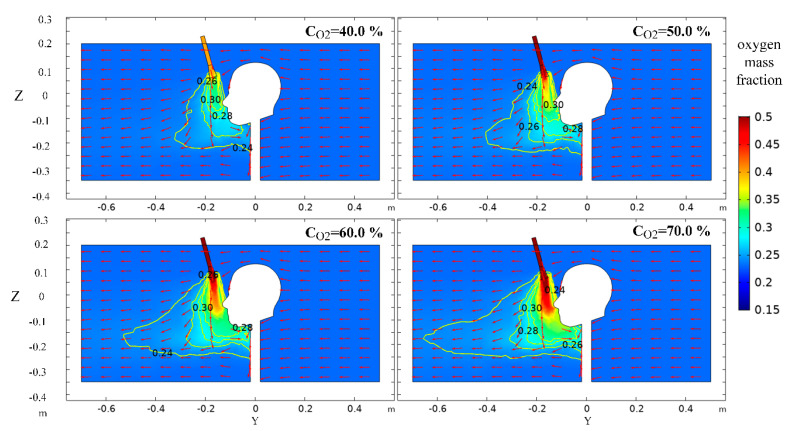
Distribution of oxygen mass fraction in X = 0 m section at different oxygen-supply concentrations.

As shown in Figure 10, with the increase of oxygen concentration, the oxygen mass fraction at the same axial distance of the oxygen-supply duct increases, and the oxygen mass fraction gradient between the nozzle outlet and the human nose decreases, while the oxygen mass fraction near the human nose increased significantly. At the same time, the diffusion range of oxygen increases significantly, which is due to convective diffusion process and concentration difference with the surrounding environment after the oxygen-rich flow enters the environment. The larger the concentration difference is, the wider the diffusion range is. The difference of the diffusion distance of oxygen in the axial direction of the oxygen-supply duct is small, which is because the outflow velocity of oxygen is the same, and the axial diffusion ability of oxygen is similar. The higher the oxygen-supply concentration is, the more oxygen can be carried by the airflow in the tunnel in the process of moving towards the front of the human body, resulting in significant differences in the radial extension distance of oxygen.

**Figure 11:**
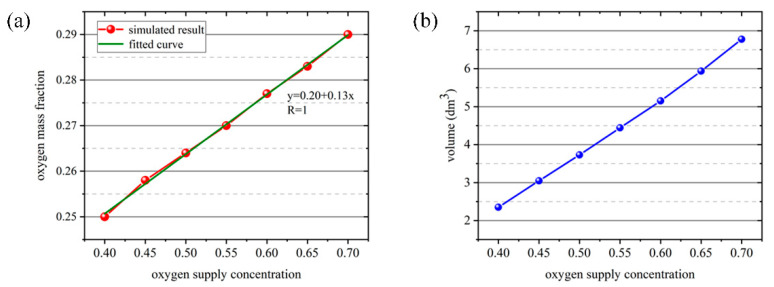
(**a**) Fitted curve of oxygen mass fraction of the air inhaled by human at different oxygen-supply concentrations. (**b**) Volumetric distribution of regions with oxygen mass fraction up to 24.35% at different oxygen-supply concentrations.

The oxygen mass fraction of the air inhaled by human at different oxygen-supply concentrations is fitted as a function, and the result was shown in Figure 11a. It can be seen from the figure that the growth curve of the oxygen concentration and the oxygen mass fraction of the air inhaled by human increases linearly, according with the first-order function relationship. As shown in Figure 11b, the volume of the region with the oxygen mass fraction reaching 24.35% gradually increased with the increase of oxygen-supply concentration, and the increasing trend was stable. Therefore, the oxygen-supply concentration has a significant effect on the stability of the individual oxygen-supply device.

### 5.3. Tunnel Airflow Velocity

The airflow field in the tunnel has a certain influence on the direction of the oxygen flow ejected from the nozzle and the mixing degree of oxygen and the air. Therefore, in addition to the influence of the parameters of the individual oxygen-supply device itself, the airflow in the tunnel is also an important factor affecting the stability of this device. In this paper, the influence of different ventilation environment on the effect of local oxygen enrichment was studied by adjusting the airflow velocity at the inlet. The tunnel airflow velocities were 0.2 m/s, 0.4 m/s, 0.6 m/s, and 0.8 m/s, respectively, and other settings were consistent with Case 3.

**Figure 12:**
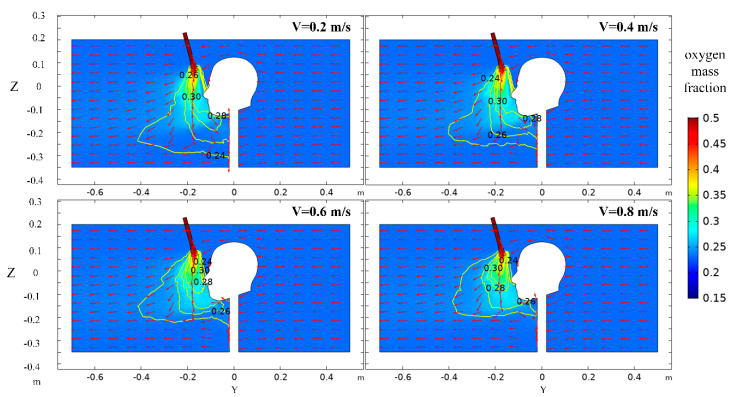
Distribution of oxygen mass fraction in X = 0 m section at different tunnel airflow velocities.

As shown in Figure 12, the higher the tunnel airflow velocity, the stronger the driving force of the tunnel airflow to the forward of the oxygen flow and the more obvious the disturbance to the oxygen flow at the outlet of the nozzle, resulting in the oxygen spreading to the front of the human body. The radial distance of the oxygen-enriched area extends ahead of the human body, while the axial distance shrinks, and the oxygen mass fraction on the axial distance is small. The increase of the air velocity at the inlet means that the air volume in the tunnel is larger, and the airflow in the tunnel absorbs more oxygen while moving to the front of the human body, resulting in the decrease of oxygen mass fraction at the human nose. Therefore, tunnel airflow has a significant impact on the stability of the local aeration device.

**Figure 13:**
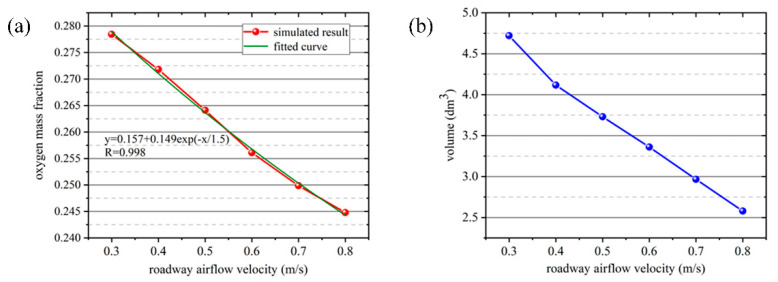
(**a**) Fitted curve of oxygen mass fraction of the air inhaled by human at different tunnel airflow velocities; (**b**) Volumetric distribution of regions with oxygen mass fraction up to 24.35% at different tunnel airflow velocities.

The oxygen mass fraction of the air inhaled by human at different inlet velocities was fitted as a function, and the result was shown in Figure 13a. It can be seen from the figure that the attenuation curve of the tunnel airflow velocity and the oxygen mass fraction of the air inhaled by human is a monotonic attenuation function, according with exponential function relationship. When the inlet air velocity increases from 0.3 m/s to 0.8 m/s, the mass fraction of human inhaled oxygen shows a decreasing trend, and the decreasing trend is slowed down. As shown in Figure 13b, with the increase of tunnel airflow velocity, the volume of the region with oxygen mass fraction reaching 24.35% showed a downward trend. With the increase of the tunnel airflow velocity, the power of the fan increases, and the more energy is consumed, resulting in a decrease in the oxygen enrichment effect of the individual oxygen-supply device. Combined with human comfort, it is necessary to adjust the best fan wind velocity to achieve a safe, comfortable, and low-energy-consuming working environment.

## 6. Influence Degree of Oxygen Enrichment Factors

The orthogonal experimental design is an efficient method for studying multi-factors and multi-level objects. Some representative points are selected from the whole to carry out the test according to the orthogonality, which can greatly reduce the workload by analyzing the representative test results to understand the overall test situation. In addition, range analysis is used to judge the degree of influence of major factors according to the range of each factor in the orthogonal test result.

Through the analysis of the above-mentioned simulation results, it was proved that the oxygen-supply velocity, oxygen-supply concentration, and the tunnel airflow velocity affected the oxygen distribution. However, the degree of the influence of these three factors on the oxygen enrichment was unknown. Therefore, the orthogonal design method was used to simulate three levels of each factor. The level selection was shown in Table 2, and the orthogonal design arrangement and test results were shown in Table 3. According to the range of each factor, the order of importance was given. The higher the range of each factor was, the more important the factor was.

The calculation using Minitab software shows that the ranges of oxygen-supply concentration, tunnel airflow velocity, and oxygen-supply velocity were 2.86, 2.63, and 1.83, respectively. It can be seen that the major influencing factors of oxygen enrichment in the tunnel of the plateau mine were, in a descending sequence, as follows: oxygen-supply concentration, tunnel airflow velocity, and oxygen-supply velocity. The difference of range between the tunnel airflow velocity and the oxygen-supply concentration was small, so the importance of these two factors was close to each other. In the practical application of the project, it is convenient to adjust the oxygen-supply concentration, and the tunnel airflow velocity is affected by the airflow in the tunnel, which is difficult to adjust and control. Therefore, when designing and optimizing the individual oxygen-supply device, the influence of tunnel wind flow on the oxygen enrichment effect should be considered. The optimal level combination was 5 m/s oxygen-supply velocity, 60% oxygen-supply concentration, and 0.2 m/s tunnel airflow velocity, which increases the oxygen mass fraction of air inhaled by human to 30.42%.

## 7. Conclusions

This paper proposed an individual oxygen-supply device, which used a nozzle to align the human nose for local oxygen-supply to solve the problem of low oxygen content in tunnel of the plateau mine and achieve a safer and more comfortable environment with lower energy consumption. A series computational studies have been conducted to evaluate the oxygen mass fraction distribution and oxygen enrichment effect after using the individual oxygen-supply device. The results of this study are summarized as follows:

(1) When the angle of the oxygen-supply tube is 15 °C and the level of the nozzle outlet is 0.06 m lower than the top of the head, the oxygen mass fraction of the air inhaled by human reaches 26.4%, and the oxygen increase effect is better than other nozzle outlet positions. The oxygen mass fraction of the air inhaled by human had a positive correlation exponential function, a positive correlation linear function, and a negative correlation exponential function, respectively, concerning oxygen-supply velocity, oxygen-supply concentration, and tunnel airflow velocity.
(2) Based on the orthogonal test results and range analysis, we found that the difference of range between the tunnel airflow velocity and the oxygen-supply concentration is small, indicating the level of importance both close, while the influence of oxygen-supply velocity was the most limited. In the practical application of the project, the adjustment of the oxygen-supply concentration is more convenient, and the wind velocity at the air inlet end is affected by the airflow of the tunnel, making it difficult to control. Therefore, when designing and optimizing the individual oxygen-supply device, the influence of tunnel airflow on the stability of the device should be mainly considered. The optimal level combination is an oxygen-supply velocity of 5 m/s, an oxygen-supply concentration of 60%, and a tunnel airflow velocity of 0.2 m/s, which increases the oxygen mass fraction of air inhaled by human to 30.42%.
(3) It is worth mentioning that, because of the complexity of the actual working environment of the mine tunnel, this paper simplified the model to a certain extent, and the simulation was carried out in a more ideal state. In future work, the airflow generated by different ventilation systems should be considered comprehensively to establish a suitable physical model and perfect boundary conditions settings, to further optimize the individual oxygen-supply device.

## Figures and Tables

**Table 1:** Boundary conditions.

Type	Parameter
Temperature	288.15 K
Atmospheric pressure	66614 KPa
Air density	0.8064 kg/m^3^
Airflow inlet	Velocity-inlet, 0.5 m/s
Airflow outlet	Outflow
Oxygen-supply duct	Velocity-inlet, 5 m/s
Nose gas inlet	Velocity-inlet, 4.5 m/s

**Table 2:** Orthogonal factor level table.

Factor Level	Oxygen-Supply Velocity (m/s)	Oxygen-Supply Concentration %	Tunnel Airflow Velocity (m/s)
1 level	3	40	0.2
2 level	5	50	0.5
3 level	7	60	0.8

**Table 3:** Orthogonal experimental results.

Test Number	Oxygen-Supply Velocity (m/s)	Oxygen-Supply Concentration (%)	Tunnel Airflow Velocity (m/s)	Oxygen Mass Fraction (%)
1	3	40	0.2	25.52
2	3	50	0.5	25.15
3	3	60	0.8	23.74
4	5	40	0.5	25.00
5	5	50	0.8	24.48
6	5	60	0.2	30.42
7	7	40	0.8	24.29
8	7	50	0.2	24.47
9	7	60	0.5	28.51
Range	1.83	2.86	2.63	
Optimal level	V_2_	C_3_	U_1_	
Optimal combination	V_2_C_3_U_1_			
